# Baiyacha, a wild tea plant naturally occurring high contents of theacrine and 3″-methyl-epigallocatechin gallate from Fujian, China

**DOI:** 10.1038/s41598-020-66808-x

**Published:** 2020-06-16

**Authors:** Ji-Qiang Jin, Chen-Kai Jiang, Ming-Zhe Yao, Liang Chen

**Affiliations:** Tea Research Institute of Chinese Academy of Agricultural Sciences, Key Laboratory of Tea Biology and Resources Utilization, Ministry of Agriculture and Rural Affairs, 9 South Meiling Road, Hangzhou, Zhejiang 310008 China

**Keywords:** Plant sciences, Secondary metabolism

## Abstract

Baiyacha (BYC) is a kind of wild tea plant growing and utilizing in the remote mountain area of Fujian province, Southeastern China. However, scientific studies on this plant remain limited. Our results showed that BYC exhibits the typical morphological characteristics of *Camellia gymnogyna* Chang, a closely related species of *C. sinensis* (L.) O. Kuntze, which was not found in Fujian before. Chemical profiling revealed that parts of BYC plants are rich in purine alkaloids and catechins, especially featuring high levels of theacrine and 3″-methyl-epigallocatechin gallate (EGCG3″Me), chemical compounds with multiple biological activities that are rarely observed in regular tea plants. The contents of EGCG3″Me and theacrine in BYC both increased with the leaf maturity of tea shoots, whereas the caffeine content decreased significantly. The obtained results provide abundant information about the morphology and chemical compounds of BYC and may be used for tea production, breeding, and scientific research in the future.

## Introduction

Aside from water, tea is the oldest and most popular non-alcoholic beverage in the world. This beverage not only exerts a profound impact on Chinese culture, health, medicine, and trade but also plays an important role in the daily lives of numerous people in Asia and the whole world^1^. The main characteristic chemicals in the tea include polyphenols, theanine, purine alkaloids, and volatiles. Catechins are the principal polyphenols, with concentrations ranging from 80 mg/g to 260 mg/g, in tea leaves^2^. In regular tea, caffeine and (−)-epigallocatechin-3-gallate (EGCG) account for more than half of the proportion of purine alkaloids and catechins, respectively^3^. Recently, theacrine (1,3,7,9-tetramethyluric acid), a rare purine alkaloid in tea, has gained wide attention for its multiple biological activities, including sedative and hypnotic activities^4^, antidepressant^5^, anti-inflammatory, and analgesic effects^6^, reduction of stress damage in liver cells^7^, and improvement of exercise capacity^8^. 3″-Methyl-epigallocatechin gallate (EGCG3″Me), an *O*-methylated EGCG, only abundantly exists in few tea plants. In recent years, several studies have shown that EGCG3″Me possesses strong antiallergic^9,10^, antihypertensive^11^, antioxidant^12^, and hepatoprotective effects^13^. As EGCG3′′Me contains a methyl group, the whole molecule is in a fat-soluble state and shows better stability and higher oral absorption rate than EGCG^14^. Therefore, a tea enriched in EGCG3′′Me and/or theacrine may exhibit significantly healthy values and substantial market potential, whereas high levels of EGCG3′′Me (>10.0 mg/g) or theacrine (>15.0 mg/g) are currently only found in limited tea germplasms. A tea plant with naturally occurring high contents of theacrine and EGCG3′′Me has not been reported yet.

Elite germplasms are the material basis for the original innovation of breeding and the strategic resources for realizing sustainable and leap-forward development of the tea industry. Wild tea plants maintained their original evolutionary characteristics due to their unique living environment and non-artificial cultivation; they also contain diverse and rare biochemical components compared with cultivated tea plants. For example, two caffeine-free tea plants in China, cocoa tea and Hongyacha (HYC), abundantly contain several compounds not detected in regular tea; these compounds include gallocatechin-(4 → 8)-gallocatechin gallate and (−)-gallocatechin-3,5-di-O-gallate^15,16^. Several wild tea plants collected from Guizhou show high catechin index (CI, ratio of dihydroxylated catechins/trihydroxylated catechins); a new allele of flavonoid 3′,5′ hydroxylase gene identified from these plants can be applied to breeding tea plants with enhanced quality^17^. As the origin of tea plant, although China has diverse tea germplasms, the most widely cultivated tea plants are *Camellia sinensis* (L.) O. Kuntze and its varieties, and wild tea plants are rarely used. Thus, as an important gene bank, wild tea plants should receive more attention from tea researchers.

**Figure 1:**
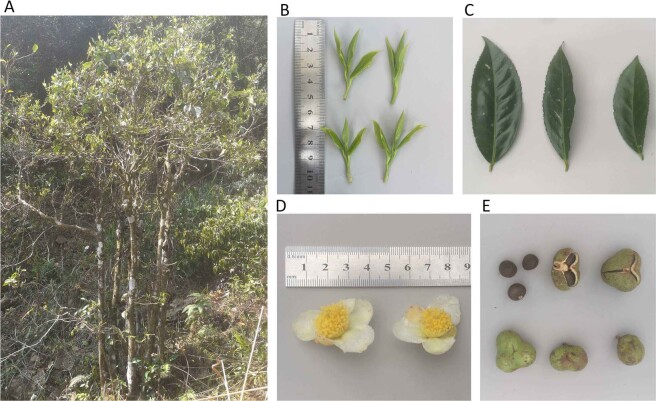
Morphological characteristic of Baiyacha: (**A**) a typical plant, (**B**) young shoots, (**C**) leaves, **(D**) flowers, and (**E**) fruits and seeds.

Fujian is the easternmost province in mainland China with wild tea plants. Baiyacha (BYC), relative to HYC with red or purple young shoots^16^, is a newly found wild tea plant with light-green young shoot in the remote mountain area in Fujian (Fig. 1). Local people feature a long history of drinking this tea for its benefit of promoting health and healing illnesses. However, the information about BYC from its various aspects, including morphology and chemical compositions, remains scant to date. Thus, in this study, the morphological characteristics of BYC were investigated to identify its botanical features, and the purine alkaloid and catechin contents in different individuals and parts of the tea shoots were analyzed to determine the chemical compositions and provide guidance for the efficient utilization of this plant in the future. The potential values of this rare resource in tea production, breeding, and scientific research were also adequately discussed.

## Results

### Morphological characteristics of BYC

**Figure 2:**
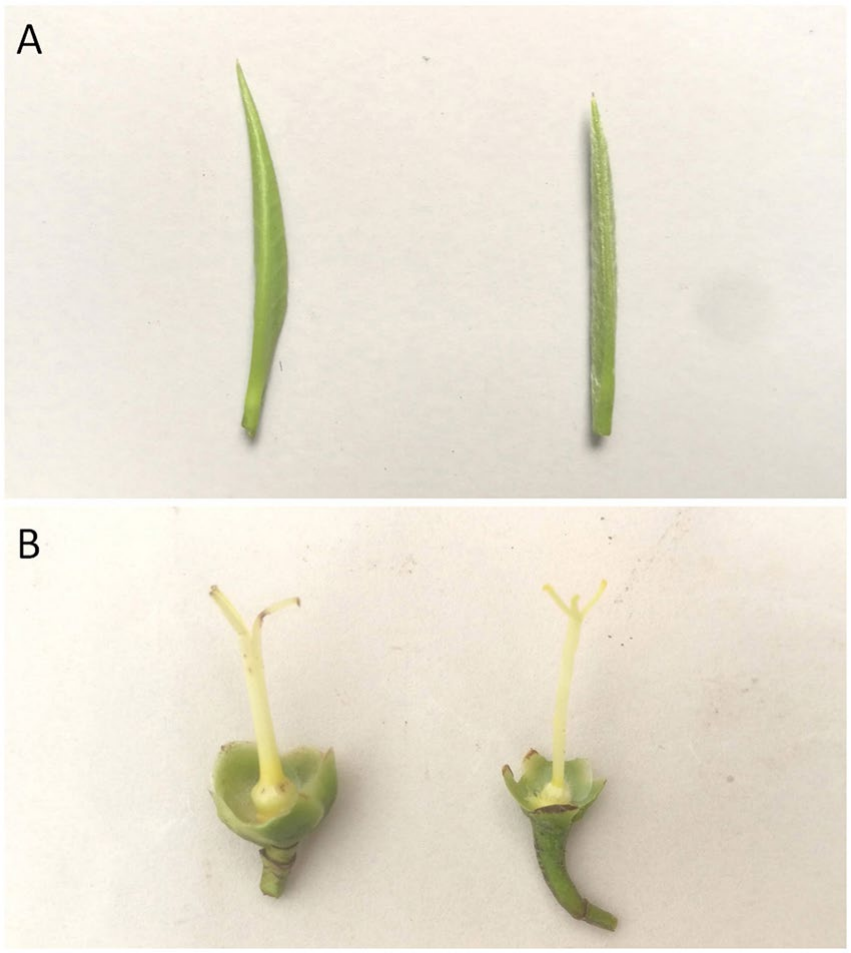
Morphological differences between Baiyacha (left) and cultivated tea plant (right): (**A**) pubescence density of bud; (**B**) pubescence density of ovary.

The plant type of BYC was semi-arbor, its growth habit was semi-upright, and density of branches was medium (Fig. 1A). In its original growing area, time of beginning of ‘one and a bud’ (one bud and one leaf) stage was varied from mid-March to late March. For young shoots, the color of second leaf at ‘two and a bud’ (one bud and two leaves) stage was light green, and pubescence density of bud was very sparse (Fig. 1B). Leaf length of BYC varied from 9.5 to 14.1 cm, and the leaf width was 3.7–4.9 cm. The shape of leaf blade was narrow elliptic, and the intensity of green color was dark (Fig. 1C). The shape in cross section of leaf blade was slightly folded upwards or flat, and texture of upper surface was moderately rugose or strongly rugose (Fig. 1C). Leaf apex shape was acuminate, and leaf base shape was acute or obtuse. The leaf margin undulation was absent or weak, and serration of margin was medium (Fig. 1C). Time of full blooming of BYC was in late October. Length of pedicels ranged from 0.4 to 0.7 cm. The flowers had five sepals. On the outer side of sepal, the pubescence and anthocyanin coloration were all absent. Flowers diameter was varied from 3.7 to 4.0 cm (Fig. 1D). Flowers of BYC had six or seven petals and petal width was 1.8–2.0 cm, and the color of inner petals was white or greenish. For most flowers, pubescence of ovary was absent (Fig. 2B). Length of style was 1.1–1.4 cm. BYC has three style splittings, and style splitting position was high (Fig. 2B). Most fruits presented globular, kidney-shaped or triangular. The pericarp thickness was 1.1–3.0 mm (Fig. 1E), and seeds appeared round. Generally speaking, BYC has distinct morphological characteristics from cultivated tea plants as showed in Fig. 2. For BYC, the pubescence of buds was very sparse, and ovary pubescence of most flowers was absent, while the buds of most cultivated tea plants were covered with dense pubescence and pubescence of ovary was present.

### High Performance Liquid Chromatography (HPLC) and Ultraperformance Liquid Chromatography–Mass Spectrometry (UPLC-MS) analyses of theacrine and EGCG3″Me

**Figure 3:**
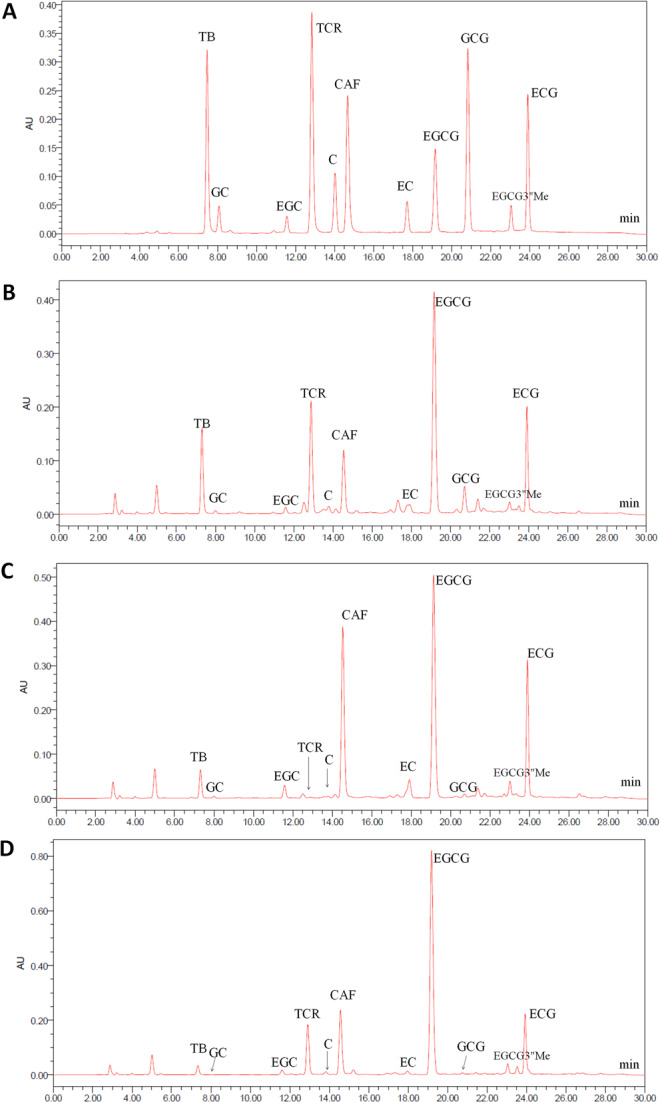
HPLC chromatograms of standard and three accessions of tea germplasms. (**A**) standard, (**B**) Ruyuan Qunti, (**C**) Benifuuki, (**D**) Baiyacha. Peak identification: TB, theobromine; GC, (+)-gallocatechin; EGC, (−)-epigallocatechin; TCR, theacrine; C, (+)-catechin; CAF, caffeine; EC, (−)-epicatechin; EGCG, (−)-epigallocatechin-3-gallate; GCG, (−)-gallocatechin-3-gallate; EGCG3″Me, 3″-methyl-epigallocatechin gallate; ECG, (−)-epicatechin-3-gallate.

**Figure 4:**
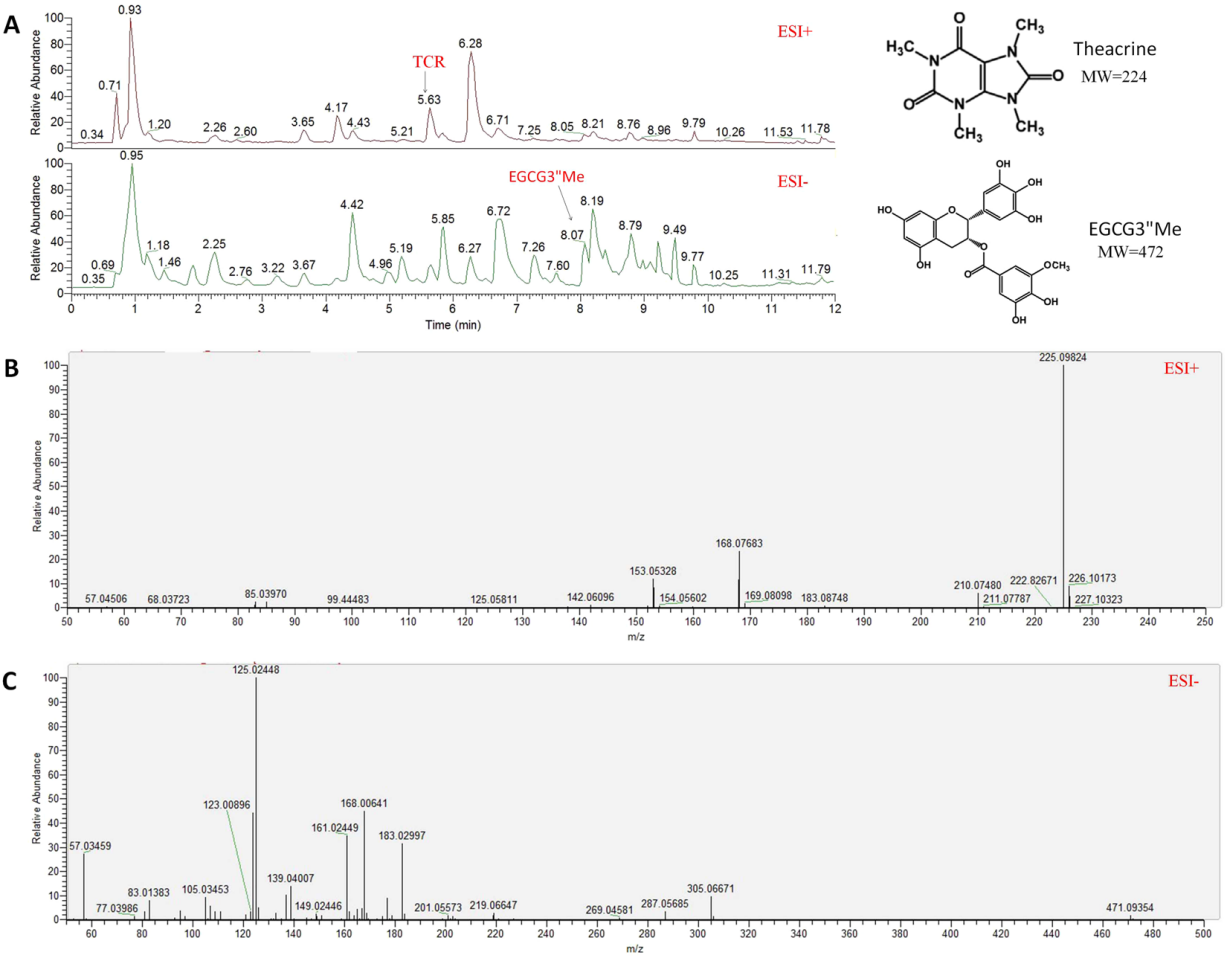
Total ion current (TIC) chromatograms and fragment ions of two compounds. (**A**) TIC of a typical Baiyacha plant in the ESI − and ESI + mode, (**B**) fragment ions of theacrine in the ESI + mode, (**C**) fragment ions of EGCG3″Me in the ESI − mode.

To determine the chemical compositions, ‘two and a bud’ young shoots of the first flush in spring were harvested from. ‘Qilan’ (QL) in BYC growing areas was used as a local control, ‘Ruyuan Qunti’ (RYQT) and ‘Benifuuki’ (BFK) were used as high-level theacrine and EGCG3″Me controls, respectively. Using our previous HPLC conditions^3^, two uncommon peaks with retention times of 12.91 and 23.05 min were observed in BYC (Fig. 3). The retention times of these compounds were consistent with those of theacrine and EGCG3″Me in RYQT and BFK, respectively. The results were preliminarily verified by using standards of theacrine and EGCG3″Me. UPLC-MS was used to further identify these compounds. As shown in Fig. 4, the peak with a retention time of 5.63 min showed a [M + H]^+^ parent ion at m/z 225.09822, and that with a retention time of 8.07 min exhibited a [M − H]^−^ parent ion at m/z 471.09354. The molecular weights of these compounds were 224 and 472, which were same as those of theacrine and EGCG3″Me, respectively. Under the MS/MS mode, the compounds with retention times of 5.63 and 8.07 min produced three major secondary fragments (MS^2^) at m/z 210.07465, 168.07681, and 153.05338 (Fig. 4B) and four MS^2^ at m/z 305.06671, 183.02997, 161.02449, and 125.02448 (Fig. 4C), which were the same as those of the authentic standards of theacrine and EGCG3″Me, respectively. Comparing the retention times, and fragmentation patterns of the two compounds in the BYC sample with those of the standards, the peaks at 5.63 and 8.07 min were identified as those of theacrine and EGCG3″Me, respectively. Then, we developed a HPLC method to determine theacrine and EGCG3″Me in the BYC samples, which yielded good linear correlation coefficients (0.9999 and 0.9987 within concentration ranges of 3.9–491.0 and 1.0–119.0 µg/mL for theacrine and EGCG3″Me, respectively), and the relative standard deviations of method repeatability were 2.20% and 1.11% (n = 5). Theacrine and EGCG3″Me were added to the tea sample for recovery experiments, and the mean values for the recoveries of them were reached 96.93% ± 1.94% (n = 3) and 113.07% ± 3.51% (n = 3), respectively.

### Chemical compositions of different BYC plants

‘two and a bud’ young shoots of 27 BYC individuals were harvested in spring and the chemical compositions of them were determined using by HPLC. Table 1 shows the specific contents of 11 compounds. The total purine alkaloids (TPA) of different BYC plants ranged from 52.5 mg/g to 76.8 mg/g, which were all higher than those of three control plants (QL, RYQT, and BFK). Among the 27 BYC individuals, the theacrine contents of 13 individuals were higher than 9.0 mg/g, and their theobromine contents were all lower than that of theacrine. The remaining 14 individuals contained low amounts of theacrine (≤1.0 mg/g). BYC11 exhibited the highest theacrine content (21.7 mg/g) among the 27 BYC individuals, similar to RYQT (23.0 mg/g). Unlike the dominant purine alkaloid in RYQT (theacrine), caffeine was the most abundant purine alkaloid at 48.0 ± 9.6 mg/g dry weight in BYC plants, and the caffeine contents of 16 BYC individuals were higher than 50.0 mg/g. For catechins, a wide variation (168.7–248.1 mg/g) in the total catechin (TC) level was observed among 27 BYC individuals, and the TC contents of 16 BYC individuals were over than 200.0 mg/g. EGCG (145.8 ± 14.3 mg/g) was the most plentiful catechin, followed by (−)-epicatechin-3-gallate (ECG) (24.2 ± 4.6 mg/g), (−)-epigallocatechin (EGC) (16.9 ± 5.7 mg/g), EGCG3″Me (10.2 ± 2.9 mg/g), and (−)-epicatechin (EC) (8.2 ± 2.0 mg/g), whereas small amounts of (+)-catechin (C) (<1.4 mg/g), (+)-gallocatechin (GC) (<1.3 mg/g), and (−)-gallocatechin gallate (GCG) (<3.2 mg/g) were detected in BYC. The EGCG3″Me content of different BYC individuals was 3.1–14.9 mg/g, and 22 BYC individuals contained higher EGCG3″Me content than BFK (7.9 mg/g).

**Table 1:** Contents of purine alkaloids and catechins in 27 Baiyacha indivduals (mg/g).

Germplasm	TB	CAF	TCR	TPA	GC	EGC	C	EC	EGCG	GCG	ECG	EGCG3″Me	TC
QL	1.3	35.7	ND	37.1	0.8	21.1	1.1	8.1	92.3	0.6	14.9	0.2	139.0
RYQT	12.9	14.1	23.0	50.0	7.0	14.5	4.7	15.1	95.7	9.9	31.2	6.2	184.4
BFK	7.3	38.6	0.3	46.3	3.5	20.0	2.6	12.6	94.3	5.4	32.1	7.9	178.4
BYC01	10.8	48.0	10.8	69.5	1.4	30.8	0.7	12.1	163.2	2.7	25.7	8.0	244.5
BYC02	6.3	36.5	16.7	59.5	0.8	22.1	ND	11.8	139.6	1.7	29.1	6.9	211.9
BYC03	10.7	50.9	15.2	76.8	0.7	14.1	ND	8.3	131.2	1.4	24.0	9.2	189.0
BYC04	11.7	39.2	16.3	67.3	0.7	13.4	ND	7.7	159.2	1.8	30.4	8.1	221.3
BYC05	10.8	51.0	0.3	62.1	ND	10.5	ND	4.9	141.2	1.3	18.5	9.5	185.9
BYC06	9.7	56.8	0.4	66.9	1.4	19.1	1.3	7.3	170.2	2.3	23.3	13.2	238.1
BYC07	4.6	53.0	0.4	58.1	1.4	18.8	ND	8.6	163.3	2.3	28.8	10.9	234.2
BYC08	10.4	58.3	1.0	69.8	ND	18.4	ND	8.0	149.5	1.1	19.5	7.1	203.6
BYC09	8.3	53.3	0.4	62.0	0.8	12.9	ND	7.5	159.0	1.3	23.8	10.8	215.9
BYC10	12.2	32.7	18.9	63.8	0.9	16.7	ND	4.8	151.8	3.2	17.9	13.9	209.3
BYC11	8.0	30.6	21.7	60.3	ND	7.4	ND	4.8	128.3	1.0	19.1	11.7	172.2
BYC12	5.3	47.0	0.6	52.8	0.8	18.4	0.5	8.6	114.1	1.1	19.6	5.6	168.7
BYC13	5.4	37.6	9.6	52.5	0.4	12.5	ND	9.7	152.1	1.5	30.3	3.1	209.6
BYC14	5.5	53.5	0.3	59.3	0.9	23.4	ND	9.6	151.1	1.6	18.4	9.8	214.6
BYC15	9.5	40.1	16.7	66.3	1.0	26.6	ND	11.6	163.0	1.3	35.0	9.6	248.1
BYC16	4.2	61.1	0.9	66.2	0.7	24.1	ND	11.6	141.7	2.6	25.7	11.0	217.4
BYC17	5.5	60.8	0.2	66.5	ND	16.3	ND	7.6	138.3	1.4	19.1	8.9	191.4
BYC18	5.1	37.4	18.4	60.8	0.8	12.7	ND	7.4	142.4	2.1	21.9	9.8	197.1
BYC19	7.7	34.4	17.6	59.7	0.7	14.1	ND	9.6	127.0	2.0	28.5	14.9	196.7
BYC20	10.5	33.4	17.9	61.8	ND	9.8	ND	7.0	122.8	1.2	23.8	10.5	175.1
BYC21	5.9	50.9	13.0	69.8	0.7	15.7	ND	6.7	133.9	1.9	28.4	11.3	198.6
BYC22	13.7	52.5	0.2	66.4	ND	10.6	ND	8.2	158.1	1.6	29.4	12.1	220.0
BYC23	7.3	51.6	11.3	70.1	ND	23.5	0.4	8.9	145.0	1.3	19.7	11.7	210.5
BYC24	4.4	57.4	0.3	62.1	0.5	13.2	ND	7.3	133.5	1.0	21.8	14.3	191.6
BYC25	9.3	52.6	0.4	62.2	ND	10.7	ND	6.2	140.4	1.2	25.3	14.1	197.9
BYC26	5.8	58.7	0.2	64.7	0.8	20.0	0.6	7.7	159.6	1.3	22.2	12.4	224.7
BYC27	11.2	56.5	0.6	68.3	0.8	21.0	ND	8.8	157.3	1.5	24.1	6.4	219.8

The values are the mean of three independent determinations. QL, ‘Qilan’; RYQT, ‘Ruyuan Qunti’; BFK, ‘Benifuuki’; BYC, ‘Baiyacha’. TB, theobromine; CAF, caffeine; TCR, theacrine; TPA, total purine alkaloids; GC, (+)-gallocatechin; EGC, (−)-epigallocatechin; C, (+)-catechin; EC, (−)-epicatechin; EGCG, (−)-epigallocatechin-3-gallate; GCG, (−)-gallocatechin-3-gallate; ECG, (−)-epicatechin-3-gallate; EGCG3″Me, 3″-methyl-epigallocatechin gallate; TC, total catechins. ND, not detected.

### Chemical compositions of different parts of tea shoots

**Figure 5:**
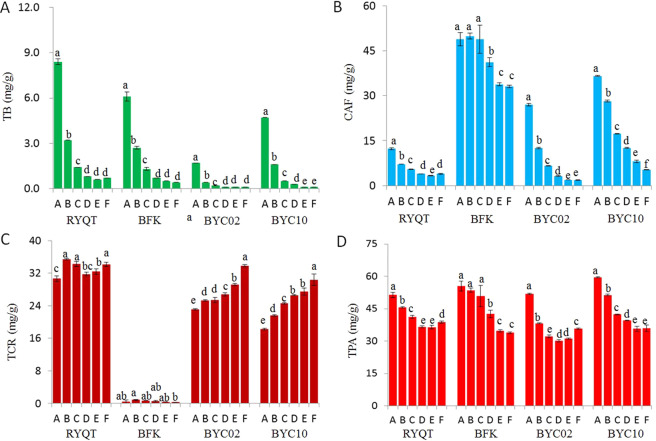
Purine alkaloid contents in different parts of ‘six and a bud’ tea shoots. (**A**) theobromine (TB), (**B**) caffeine (CAF), (**C**) theacrine (TCR), (**D**) total purine alkaloids (TPA). RYQT, ‘Ruyuan Qunti’; BFK, ‘Benifuuki’; BYC, ‘Baiyacha’. A. bud and first leaf; B. second leaf; C. third leaf; D. fourth leaf; E. fifth leaf; F. sixth leaf. The data are presented as the mean ± standard error of three independent analyses. Different letters above columns denote the existence of significant difference (p < 0.05).

**Figure 6:**
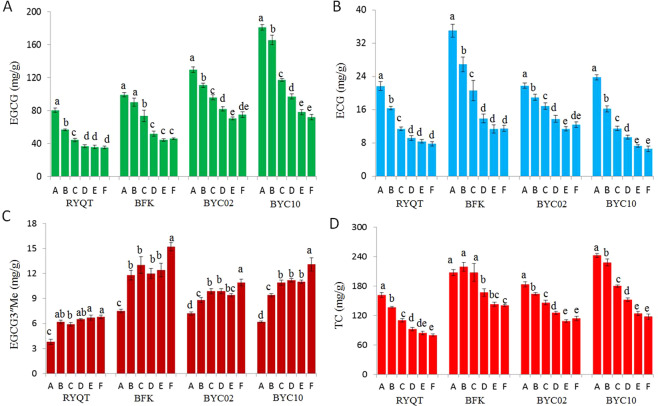
Catechin contents in different leaf position of ‘six and a bud’ tea shoots. (**A**) (−)-epigallocatechin-3-gallate (EGCG), (**B**) (−)-epicatechin-3-gallate (ECG), (**C**) 3″-methyl-epigallocatechin gallate (EGCG3″Me), (**D**) total catechins (TC). RYQT, ‘Ruyuan Qunti’; BFK, ‘Benifuuki’; BYC, ‘Baiyacha’. A. bud and first leaf; B. second leaf; C. third leaf; D. fourth leaf; E. fifth leaf; F. sixth leaf. The data are presented as the mean ± standard error of three independent analyses. Different letters above columns denote the existence of significant difference (p < 0.05).

Figure 5 shows the contents of purine alkaloids in different parts of young shoots. The theobromine and caffeine contents decreased with leaf maturity from the bud and first leaf to the sixth leaf in the four tea plants, especially in three plants containing high levels of theacrine (RYQT, BYC02 and BYC10). The sixth leaf with the highest maturity showed the least amount of caffeine (≤5.3 mg/g) (Fig. 5B). The theacrine contents in the different parts of RYQT shoots were ordered as follows: second leaf > third leaf > sixth leaf > fifth leaf > fourth leaf > bud and first leaf. The contents in the different parts of RYQT varied from 30.7 ± 0.7 mg/g to 35.4 ± 0.2 mg/g, whereas two BYC individuals (BYC02 and BYC10) presented a notably increasing trend with the leaf maturity from the bud and first leaf to the sixth leaf (Fig. 5C). In addition, the TPA presented a similar trend with theobromine and caffeine, and its contents were the highest in the bud and first leaf (Fig. 5D). Figure 6 displays the catechin contents in different parts of tea shoots. In general, the EGCG3″Me contents showed an increasing trend with the leaf maturity from the bud and first leaf to the sixth leaf to a certain extent, and the content of EGCG3″Me in the bud and first leaf was lower (p < 0.05) than that of other parts of the tea shoots, whereas EGCG, ECG, and TC levels in the different stages of leaf maturity presented a reverse trend.

## Discussion

In this study, BYC showed with distinct morphological characteristics compared with cultivated tea plants (Fig. 2). Although different taxonomic systems of Section *Thea* (L.) Dyer in genus *Camellia* were proposed according to the morphologic descriptors^18–20^, *C. gymnogyna* Chang (a closely related species of *C. sinensis*) all had the same typical morphological characteristics, including the extremely sparse pubescence of buds, three splittings of style, and absence of ovary pubescence. Our results showed that BYC exhibits these typical morphological characteristics of *C. gymnogyna* Chang. So BYC may belong to *C. gymnogyna*, which only thrived in regions of South and Southwest China, such as Guizhou, Yunnan, Sichuan, and Guangxi provinces, before. BYC is a new kind of *C. gymnogyna*, which not been reported in Fujian, an easternmost province with distributed wild tea plants in the Chinese mainland. Although with similar morphological characteristics, the chemical compositions vary substantially among the individuals in *C. gymnogyna* distributed in different growing areas. For example, the *C. gymnogyna* from Guangxi contain low content of theacrine (5.8 − 9.1 mg/g)^21^, and those from Guizhou feature a high CI trait^17^. In this study, most BYC individuals naturally contained abundant EGCG3″Me (>8.0 mg/g) and parts of BYC individuals contained high contents of theacrine (>9.5 mg/g).

In areas originally growing BYC, local people believe that BYC tea offers beneficial effects on the prevention or healing of illnesses. In the 403 accessions of spring samples previously identified using HPLC, three accessions exhibited high TC content (>200 mg/g), whereas none showed a high caffeine content (>50.0 mg/g)^3^. In this study, the caffeine and TC contents of QL, a local tea cultivar in Fujian, are only 35.7 and 139.0 mg/g, respectively. Among the 27 BYC individuals, the caffeine and TC contents of 10 individuals were all higher than 50.0 and 200.0 mg/g, respectively. Theacrine, a rare purine alkaloid in tea first reported in 1937^22^, was not widely known until its discovery in a bitter-tasting wild tea plant originating from Yunnan^23^. Tea germplasms naturally containing theacrine are also found in the morphologically diverse wild tea plants collected from Guizhou, Guangxi, Guangdong, and Jiangxi provinces, which all exhibit bitter taste. On the other hand, the green tea made from several BYC individuals shows good sensory quality with slight bitterness. Among the 27 BYC individuals, BYC11 (21.7 mg/g in ‘two and a bud’ spring sample) showed almost similar content with RYQT (23.0 mg/g), a wild tea plant with the highest contents of theacrine conserved in China National Germplasm Hangzhou Tea Repository (CNGHTR). EGCG3″Me is abundant in a limited number of tea germplasms. Only four tea cultivars with high EGCG3″Me contents (>10.0 mg/g) were observed among the 71 tea cultivars collected in China^24^. BFK, a famous tea cultivar rich in EGCG3″Me, the content of spring ‘two and a bud’ young shoots was 7.9 mg/g in this study. However, among the 27 BYC individuals, 14 contained high EGCG3″Me content (>10.0 mg/g), and 4 showed high levels of both theacrine (>15.0 mg/g) and EGCG3″Me (>10.0 mg/g) (Table 1). Moreover, the BYC plants were abundantly scattered in the vast mountain region in their growing area, indicating that the unique plants with higher contents of theacrine and EGCG3″Me would be screened out. The obtained results suggest that BYC with enriched common catechins and purine alkaloids, together with theacrine and EGCG3″Me as potent health components, could be a rare plant resource for the production of natural tea beverages. Moreover, theacrine is derived from adenosine via caffeine through two reactions, and EGCG3″Me is synthesized from EGCG catalyzed by *O*-methyltransferase (OMT); *S*-adenosylmethionine acts as a methyl donor^25,26^. Theacrine and EGCG3″Me were undetected or present in negligible amounts in most cultivated tea plants, implying that BYC may possess unique regulation mechanisms or genes encoding enzymes for theacrine and EGCG3″Me synthesis and the potential use in cultivating genetically modified tea plant enriched with theacrine and EGCG3″Me.

To effectively utilize these rare resources, the contents of purine alkaloids and catechins in BYC leaves with different levels of maturity were analyzed. The contents of caffeine and theobromine in four tea germplasm accessions all showed decreasing trends with leaf developmental stages (Fig. 5); this result might be due to the markedly declined amounts of tea caffeine synthase gene transcript during leaf development^27^. Results show that the declining rates of caffeine in BYC02 and BYC10 were faster than that of BFK, and such situation might account for the chemical conversion of caffeine into theacrine. Our results show that EGCG, ECG, and TC are generally enriched in younger BYC leaves. By contrast, the level of EGCG3″Me in tea shoots decreased with maturity (Fig. 6). This result may be due to the remarkably decreased expression levels of most genes involved in catechin synthesis during leaf maturation^28^. However, the level of *OMT* transcript involved in EGCG3″Me synthesis increased first and then decreased with leaf maturity (unpublished data).

This study provides useful information on the morphology and chemical components of BYC and guidance for its production, breeding, and scientific research. First, individuals with high contents of theacrine and EGCG3″Me, which both possess multiple biological activities, would be used as raw materials for producing special tea beverages or extracting functional components. Second, the contents of theacrine and EGCG3″Me in BYC were enriched with the leaf maturity of young shoots, whereas caffeine content decreased significantly. Oolong tea made from mature BYC leaves may feature high levels of theacrine and EGCG3″Me but contain low contents of caffeine. Fujian is one of the main producing areas of Oolong tea. Thus, making Oolong tea may be a more suitable processing method for BYC. BYC is an elite kind of tea germplasm for breeding cultivars that are rich in functional components to meet the development needs of tea industry. After introducing the beneficial genes of BYC into cultivated tea plants, the offspring will present high levels of theacrine and EGCG3″Me and low caffeine content for the longer metabolic pathways of purine alkaloids and catechins compared with the regular tea cultivars. Consumers would prefer this kind of healthy tea. Furthermore, tea plant is generally believed to have originated from Southwestern China, whereas BYC together with HYC, another wild tea plant, is also newly discovered in Fujian^16^; both in remote areas and in a state of natural isolation show disparate morphological characteristics, biochemical profile, or genes compared with various other tea plants. These special wild tea plants that are naturally distributed in Southeastern China would provide unique research materials for the origin and evolution of tea plants.

## Methods

### Plant materials

BYC plants scattered in a mountain region at altitudes of 750 − 950 m in Fujian were used as research plant materials. ‘Qilan’ (QL), an improved tea cultivar widely cultivated in Fujian, was planted in the same location and used as local control. ‘Ruyuan Qunti’ (RYQT), which features high contents of theacrine, and ‘Benifuuki’ (BFK), a famous tea cultivar rich in EGCG3″Me from the CNGHTR located in the Tea Research Institute, Chinese Academy of Agricultural Sciences (Hangzhou), were used as high-level theacrine and EGCG3″Me controls, respectively. ‘Two and a bud’ of the first flush shoots were picked from 27 BYC individuals and three control plants in spring to determine their chemical compositions. Young tea shoots were fixed with hot air at 120 °C for 5 − 8 min and then fully dried at 75 °C. The dried samples were kept frozen (−20 °C) until analysis.

In July, tea leaves of four tea germplasm accessions rich in theacrine or EGCG3″Me (RYQT, BFK, BYC02, and BYC10) collecting from their original regions and growing in CNGHTR were picked from different parts of the ‘six and a bud’ (a bud and six leaves) by hands. Each leaf represented a different level of leaf maturity. After collection, the samples were quickly dried as per previous preparation and for further analysis.

### Investigation of morphological characteristics

The morphological characteristics of BYC were described and measured on the basis of the International Union for the Protection of New Varieties of Plants (UPOV) Distinctness, Uniformity and Stability Test Guidelines for tea plant (TG/238/1)^29^. Most of the morphological characteristics, including plant type, growth habit, density of branches, leaf blade, flower, and fruit, were described and measured in November, whereas the young shoots were investigated in March.

### Chemicals and standards

The standard substances of seven catechins (EGCG, ECG, EGC, EC, GC, C, and GCG) and two purine alkaloids (theobromine and caffeine) were bought from Sigma–Aldrich Shanghai Trading Co. Ltd (Shanghai, China). EGCG3″Me was bought from Beijing Greenherbs Science and Technology Development CO., Ltd (Beijing, China). Theacrine was purchased from Shanghai Jinsui Bio-Technology Co. Ltd (Shanghai, China).

### Extraction and HPLC condition

Sample extraction and HPLC conditions were the same as those described in our previous study^3^. The TC content was calculated as the sum of C, EC, ECG, EGC, EGCG, EGCG3″Me, GC, and GCG, and TPA content was the sum of caffeine, theacrine, and theobromine.

### UPLC-MS conditions

Chromatographic separations were performed at 40 °C on a UPLC system (Ultimate 3000, Dionex, U.S.A.) equipped with a C18 column (1.8 µm, 2.1 × 100 mm, Agilent, U.S.A.) applying water with 0.1% formic acid in water (v/v) and 0.1% formic acid in acetonitrile (v/v) as binary gradients A and B, respectively, at a flow rate of 0.3 mL/min: 1–6 min, linear gradient from 5% to 20% B; 6–10 min, linear gradient to 95% B; 10–11.5 min, linear gradient to 5% B; and 11.5–15 min, 5% B. The corresponding changes in A were made. The injection volume was 2 µL.

Standards and metabolite detection was conducted on Q-Obitrap-MS (Thermo Fisher Scientific, U.S.A.). The mass spectrometer was operated in both positive and negative modes with electron spray ionization (ESI) at capillary voltage 3.50 and 3.20 KV, respectively. The temperatures of drying gas and aux gas were 320 °C and 350 °C, respectively. The full MS scan mode ranged from m/z 70 to m/z 1000 at a resolution of 70000, and the top 10 peak areas were selected. This process was followed by tandem MS (MS/MS) scanning at a resolution of 17500 with 15%, 30%, and 60% normalized collision energy, 4.0 m/z isolation window, 10 loop counts, and 10.0 s dynamic exclusion. Quality control (QC) samples were prepared by mixing samples of equal amount, with every 10 samples injected with one QC to monitor the instrument performance.

### Statistical analysis

The results were analyzed by using SPSS software (SPSS, Chicago, IL). The statistical significance of differences was determined with Tukey’s test. Significance was established at p < 0.05.
